# Analysis of Complement *C3* Gene Reveals Susceptibility to Severe Preeclampsia

**DOI:** 10.3389/fimmu.2017.00589

**Published:** 2017-05-29

**Authors:** A. Inkeri Lokki, Tea Kaartokallio, Ville Holmberg, Päivi Onkamo, Lotta L. E. Koskinen, Päivi Saavalainen, Seppo Heinonen, Eero Kajantie, Juha Kere, Katja Kivinen, Anneli Pouta, Pia M. Villa, Leena Hiltunen, Hannele Laivuori, Seppo Meri

**Affiliations:** ^1^Immunobiology, Research Programs Unit, University of Helsinki, Helsinki, Finland; ^2^Medical and Clinical Genetics, University of Helsinki and Helsinki University Hospital, Helsinki, Finland; ^3^Bacteriology and Immunology, University of Helsinki, Helsinki, Finland; ^4^Clinic of Infectious Diseases, HYKS Inflammation Center, Helsinki University Hospital, University of Helsinki, Helsinki, Finland; ^5^Department of Biosciences, University of Helsinki, Helsinki, Finland; ^6^Department of Obstetrics and Gynecology, Helsinki University Hospital, University of Helsinki, Helsinki, Finland; ^7^Chronic Disease Prevention Unit, Department of Health, National Institute for Health and Welfare, Helsinki, Finland; ^8^Children’s Hospital, Helsinki University Hospital, University of Helsinki, Helsinki, Finland; ^9^PEDEGO Research Unit, MRC Oulu, Oulu University Hospital, University of Oulu, Oulu, Finland; ^10^Department of Biosciences and Nutrition, Karolinska Institutet, Huddinge, Sweden; ^11^Folkhälsan Institute of Genetics, Helsinki, Finland; ^12^Molecular Neurology, Research Programs Unit, University of Helsinki, Helsinki, Finland; ^13^Division of Cardiovascular Medicine, University of Cambridge, Cambridge, UK; ^14^Department of Government Services, National Institute for Health and Welfare, Helsinki, Finland; ^15^Finnish Red Cross Blood Service, Helsinki, Finland; ^16^Institute for Molecular Medicine Finland, HiLIFE, University of Helsinki, Helsinki, Finland

**Keywords:** preeclampsia, complement, *C3*, association study, gene regulation, genetic risk, pregnancy complication, innate immunity

## Abstract

Preeclampsia (PE) is a common vascular disease of pregnancy with genetic predisposition. Dysregulation of the complement system has been implicated, but molecular mechanisms are incompletely understood. In this study, we determined the potential linkage of severe PE to the most central complement gene, *C3*. Three cohorts of Finnish patients and controls were recruited for a genetic case-control study. Participants were genotyped using Sequenom genotyping and Sanger sequencing. Initially, we studied 259 Finnish patients with severe PE and 426 controls from the Southern Finland PE and the Finnish population-based PE cohorts. We used a custom-made single nucleotide polymorphism (SNP) genotyping assay consisting of 98 SNPs in 18 genes that encode components of the complement system. Following the primary screening, *C3* was selected as the candidate gene and consequently Sanger sequenced. Fourteen SNPs from *C3* were also genotyped by a Sequenom panel in 960 patients with severe PE and 705 controls, including already sequenced individuals. Three of the 43 SNPs observed within *C3* were associated with severe PE: rs2287845 (*p* = 0.038, OR = 1.158), rs366510 (*p* = 0.039, OR = 1.158), and rs2287848 (*p* = 0.041, OR = 1.155). We also discovered 16 SNP haplotypes with extreme linkage disequilibrium in the middle of the gene with a protective (*p* = 0.044, OR = 0.628) or a predisposing (*p* = 0.011, OR = 2.110) effect to severe PE depending on the allele combination. Genetic variants associated with PE are located in key domains of C3 and could thereby influence the function of C3. This is, as far as we are aware, the first candidate gene in the complement system with an association to a clinically relevant PE subphenotype, severe PE. The result highlights a potential role for the complement system in the pathogenesis of PE and may help in defining prognostic and therapeutic subgroups of preeclamptic women.

## Introduction

Preeclampsia (PE) is a serious vascular complication of pregnancy, which may lead to a life-threatening multi-organ dysfunction and a convulsive condition, eclampsia (1). PE affects 3–5% of pregnancies in all ethnic groups. The development and progression of the disease are unpredictable with delivery being the only effective cure.

Preeclampsia has been the subject of numerous genetic studies and several associating single nucleotide polymorphisms (SNPs) have been identified. Among the genes where associating SNPs have been described are genes linked to hypertension and vascular and metabolic disease (2–4), all diseases whose risk is increased in the later life of PE patients (5). Furthermore, genes encoding for proteins involved in the immunological processes have also been found to harbor SNPs that predispose patients to PE (6, 7).

Pregnancy is the ultimate immunological paradox, where the maternal immune system must accommodate to protect the mother and growing fetus from pathogens while allowing the semiallograft fetus to persist and thrive. PE is a vascular disease that involves poor placentation (8), especially in the severe and/or early onset (diagnosis or delivery <34 weeks of gestation) forms of the disease, where immunological mechanisms have been implicated (9, 10). Among the immunological effector mechanisms, inadequate control of the maternal complement system has been suggested to contribute to the etiology of PE (11, 12).

The complement system is a part of the immune system that is involved in generating inflammation and mediating the clearance of microbes and injured tissue materials. It can be activated by the classical, the lectin, or the alternative pathway, which proceed stepwise in a controlled cascade of interactions between surface-bound and soluble proteins in the serum. Complement C3 is the central component of all activation pathways. It is among the most ancient components of innate immunity that has evolved over 1,000 million years ago (13). Indeed, the functional domains of the human C3 are conserved in corals and Cnidarians (14, 15). The ancient evolutionary attribute of C3 and its abundance in the human serum indicate its important role as the key component of immunity against infection and in the discrimination between self and non-self (16).

C3 is a large protein formed by pair of disulfide-linked α- and β-chains and 13 individual domains. In shape, it is a “robot”-like molecule that has eight macroglobulin domains “the body,” a linker (LNK), the C3a anaphylatoxin, an arm-like region with the “C1r/s, UEGF, BMP1” (CUB), a thioester-containing domain (TED), an N-terminal domain (α'NT), and the “head” (C345C) linked to the body with an anchor (17). The domains are encoded by 41 exons of the *C3* gene. When C3 is activated to C3b, an internal thioester bond is disrupted allowing covalent attachment of C3b to target surfaces. Subsequently, factor B binds to the MG2 and CUB domains of C3b (18). A C3 convertase, C3bBb, is formed when factor D activates C3b-bound factor B to breakdown product. Thereafter, C3bBb cleaves new C3 molecules to C3b to release anaphylatoxic C3a fragments to the circulation (19). The main inhibitors of C3 activation, factor H, decay accelerating factor (CD55), and CR1 bind partially to the factor B-binding site to prevent or disrupt C3bBb formation (17, 20).

Mao et al. showed that alternative pathway complement activation is the key mechanism for reproductive failure in complement inhibitor deficient (*Crry*^−/−^) mice (21). Recently, it was shown that alternative complement pathway becomes activated also in human pregnancies, where severe PE develops (11). Successful treatment of a patient suffering from HELLP syndrome (hemolysis, elevated liver enzymes, low platelet count), a life-threatening complication of PE, by eculizumab, a targeted inhibitor of complement protein C5, demonstrated that the complement system could provide a promising target for drug development in severe PE (22). C5 is the initiator of the final stages of complement activation, i.e., the lytic terminal pathway.

We have looked for SNP association with severe PE among 18 genes coding for the complement system. The most promising associations were found in *C3*, where linkage both to individual SNPs and to a distinct haplotype, was observed. *C3* was thus subsequently chosen for detailed capillary sequencing of its exons and promoter regions (PROMs) in women with severe PE and controls with non-PE pregnancies.

## Materials and Methods

**Figure 1:**
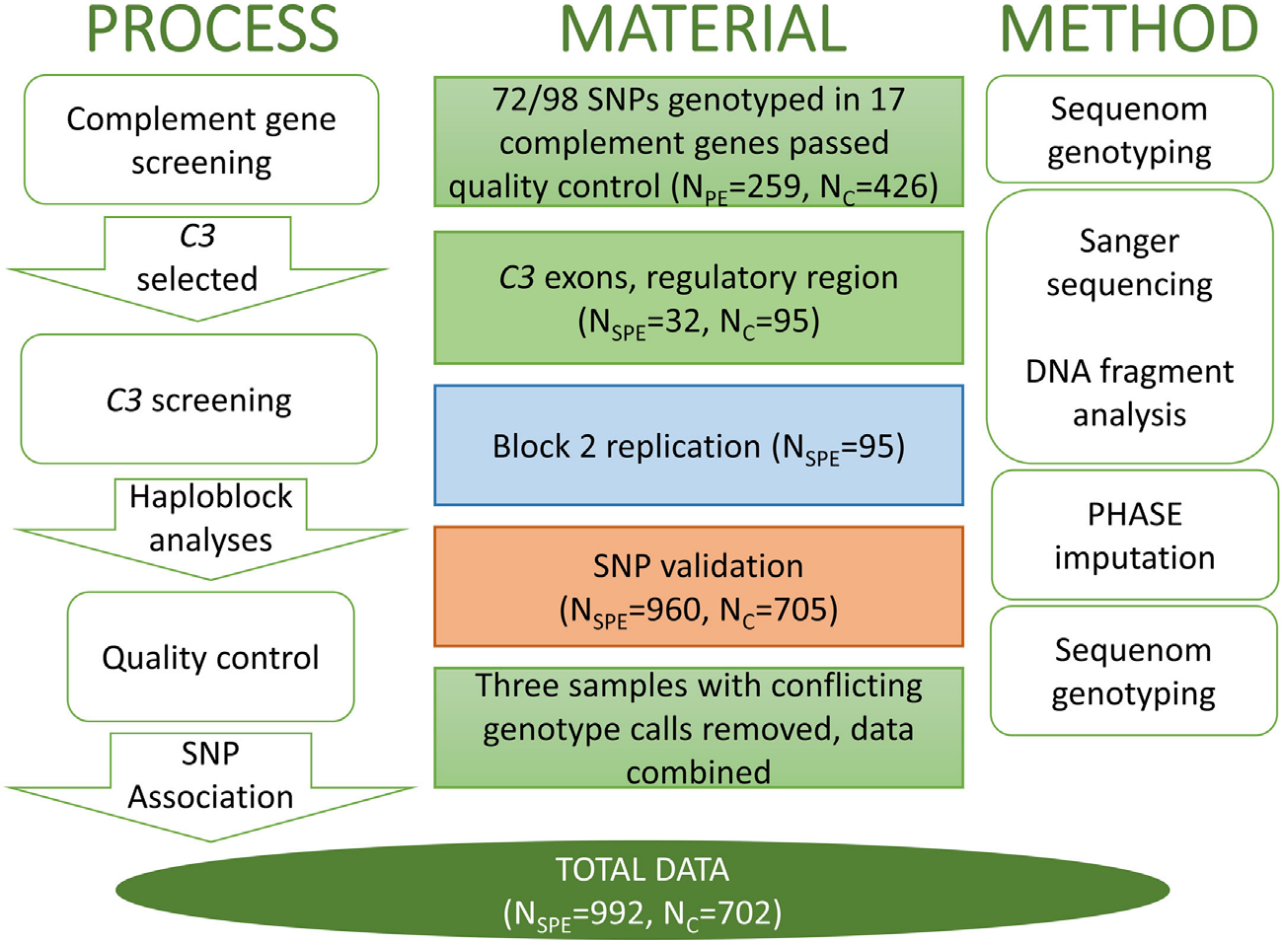
**The workflow of C3 project with the analysis process and appropriate method in parallel**. The single nucleotide polymorphisms (SNPs) that were assessed in each stage are identified by the respective color in the method panel in Figure 2. SPE, severe preeclampsia.

This case-control study was conducted at the Department of Medical and Clinical Genetics and Institute for Molecular Medicine Finland in the University of Helsinki. The number of women and methods of genotyping in each stage of the study are described in detail in the work flow chart (Figure 1). All subjects provided a written informed consent in accordance with the Declaration of Helsinki. Study protocols were approved by the local Ethical Committees, specifically, for the FINNPEC study, ethical approval has been obtained from the Coordinating Ethics Committee, Hospital District of Helsinki, and Uusimaa, for the Finnish population-based PE cohort was approved by the ethics committee of the Finnish Red Cross Blood Service and by the Ministry of Social Affairs and Health and for Southern Finland PE study was approved by the Ethics Committee of the Department of Obstetrics and Gynaecology at Helsinki University Central Hospital.

### Selection of Study Subjects and Diagnostic Criteria

Study subjects were selected from three Finnish PE cohorts; The Finnish population-based PE cohort (23), The Southern Finland PE cohort (23), and the Finnish Genetics of PE Consortium (FINNPEC) cohort (24) (Table S1 in Supplementary Material). Only women with a singleton pregnancy were included in the study. Except for three women from the Southern Finland PE cohort, where data on subjective symptoms were not available, all women with PE met the criteria of severe PE (23). Severe PE was defined as having repeatedly maximum systolic blood pressure (BP) ≥ 160 mmHg and/or maximum diastolic BP ≥ 110 mmHg or proteinuria ≥5 g/day or significant subjective symptoms in a woman diagnosed with PE according to the American College of Obstetricians and Gynecologists (25).

The clinical characteristics of all women whose samples were used for sequencing and Sequenom genotyping are described in Table 1. Body mass index was defined as the pre-pregnancy weight in kilograms divided by height in meters squared (kg/m^2^). Pregnancy weight and height measures were obtained from the antenatal charts. Relative birth weight SD units (*Z*-score) were calculated according to Finnish standards (26).

**Table 1:** **Clinical characteristics of the Southern Finland cohort (*N* = 32, data missing for five individuals) and the Finnish Genetics of Preeclampsia (FINNPEC) cohort participants in *C3* sequencing and genotyping**.

	Controls (*N* = 702)	Severe preeclampsia (PE) (*N* = 991)	*p*-Value* (compared to controls)	Severe PE	*p*-Value* (compared to controls)
				The Sanger sequencing stage (*N* = 27) median (25th, 75th percentile)	
Age, years	29.5 (26, 33)	31 (27, 35) *N* = 986	<0.001	31 (25, 33)	0.742
Pre-pregnancy body mass index, kg/m^2^	23 (20.8, 25.9)	24 (21.3, 28) *N* = 988	<0.001	22.5 (20.7, 24)	0.132
Highest systolic blood pressure (BP), mmHg	125 (118, 133)	171 (161, 184)	<0.001	170 (160, 180)	<0.001
Highest diastolic BP, mmHg	82 (78, 87)	112 (107, 118)	<0.001	105 (100, 110)	<0.001
Proteinuria (DU-prot, diurnal collection sample), g/d		4.2 (1.8, 7.1) *N* = 927	NA	5 (1.7, 15.2)	0.002
Proteinuria (U-prot, single sample), g/l, median (max, min)^a^		1.3 (0.7, 3.2), *N* = 13	0.189	NA	NA
Proteinuria measured by dipstix *N* (% positive: +, ++, +++)^b^	23 (3)	54 (5.4)	0.03	NA	NA
Primipara *N* (%)	377 (54)	733 (73)	<0.001	31 (100)	0.017
Birth weight SD score	0 (−0.6, 0.7) *N* = 700	−1.3 (−2.0, −0.5) *N* = 990	<0.001	−1.7 (−2.3, −0.7)	<0.001
Gestational age at birth, weeks	40 (39, 41)	37 (34, 38)	<0.001	36 (31, 38)	<0.001

*Mann–Whitney *U* (continuous) or χ^2^ (categorical), Fisher’s exact for small groups *N* < 5.

*^a^Among those with no diurnal protein available*.

*^b^Among those with no quantitative (diurnal or single sample) protein measurement*.

*Values for continuous variables are median (25, 75 percentiles) unless otherwise indicated, the number of subjects is indicated where data are not available for all participants*.

*Proteinuria was also observed in 11 controls, who did not meet the diagnostic criteria of PE*.

#### Subjects in Complement Genotyping

The SNP genotyping was performed in 259 PE women and 426 non-PE controls. The PE women and controls were selected from the Finnish population-based PE cohort and the Southern Finland PE cohort with preference on severity of the disease.

#### Subjects in *C3* Sequencing and Microsatellite Analysis

We performed *C3* sequencing and the microsatellite analysis of the upstream regulatory motif in 32 severe PE women from the Southern Finland PE cohort (Table 1) and 95 non-PE controls from the FINNPEC cohort. These data were also used in the relative extended haplotype homozygosity (REHH) statistics.

#### Subjects in *C3* Replication by Sequencing

**Figure 2:**
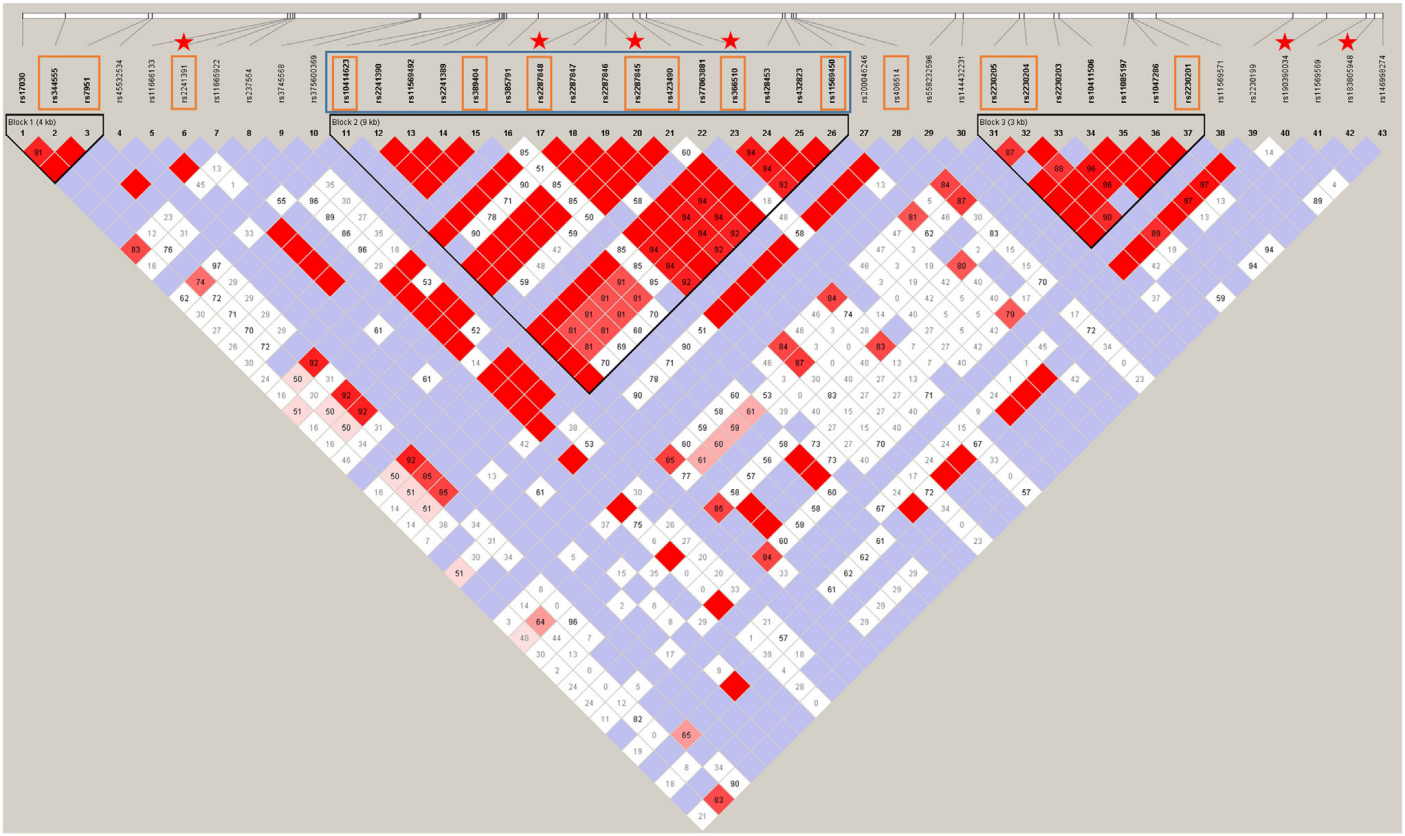
**The haploblock structure within *C3* determined by Sanger sequencing and replication studies**. The intensifying gradient of red represents the increasing relative linkage disequilibrium between two variants. The associating Block 2 is identified by the black triangle in the middle of the gene and had a multiallelic D' score of 0.623. The single nucleotide polymorphisms (SNPs) that were assessed in each replication stage are identified by the respective color square that correspond to colors in the material panel in Figure 1. The independently associating SNPs are indicated by stars. All SNPs in this image are listed in Table S4 in Supplementary Material.

Ninety-five women with severe PE from the FINNPEC cohort were selected for the second stage of sequencing, which involved re-sequencing the middle part of the gene indicated by blue in Figure 2. The replication sequencing data were used in the REHH statistics.

#### Subjects in Replication by Sequenom Genotyping

Fourteen SNPs were validated in 960 women with severe PE and 705 non-PE controls from the FINNPEC cohort (including all of the FINNPEC participants included in the initial exploratory Sequenom genotyping). These data were combined with sequenced genotypes of *C3* in the initial phase and replication phase for the single SNP association analysis. The study subjects are described in Table 1.

### Complement Genotyping

A custom SNP genotyping was performed using Sequenom’s MassARRAY MALDI-TOF Mass Spectrometry Compact platform and iPLEX Gold chemistry (Sequenom Inc., San Diego, CA, USA) with standard protocols as described elsewhere (27). Briefly, 18 genes coding for components of the complement system were chosen for genotyping and for each gene, SNPs with assumed relevance were selected based on published data on protein function, activity, or disease association (Table S2 in Supplementary Material). Furthermore, we included potentially functional nonsense and missense SNPs, and finally, we also included some intronic, promoter or 3' end SNPs as markers of association. A total of 98 SNPs were assayed in four multiplexes of 15–34 markers each. We focused on SNPs with minor allele frequencies >0.05 in European populations based on the HapMap data (28). Genotypes were analyzed using Sequenom’s MassARRAY Typer version 4.0 software.

### Microsatellite Analysis

The size of the (CA)_n_ repeat polymorphism GF100472 in the promoter area of the *C3* gene was determined by fragment analysis. The repeat area was amplified by PCR using FAM-labeled forward primer and non-labeled reverse primer (Table S3 in Supplementary Material) in PCR conditions of an initial denaturation at 95°C for 10 min, followed by 32 amplification cycles of 95°C for 30 s, 67°C for 30 s, and 72°C for 50 s and final extension at 72°C for 7 min. The sizes of the amplified fragments were determined using an automated capillary sequencer ABI3730xl DNA Analyzer (Applied Biosystems). GeneScan 500 LIZ Size Standard (Applied Biosystems) was used to size the fragment data. The number of CA repeats was determined with Gene Mapper v4.0 software (Applied Biosystems) and the repeat alleles were classified as short length (CA_8–10_), medium length (CA_11–12_), or long (CA_15_) for the purpose of analysis.

### *C3* Sequencing

The exonic areas of the *C3* gene including flanking intronic regions and potential splice sites and 650 kb PROM were sequenced using standard Sanger sequencing with primers detailed in S3. Amplitaq Gold (Applied Biosystems) enzyme was used in the reactions. Samples were purified from excess primers by digestion using 2.5 U of Shrimp Alkaline Phosphatase USB and 5 U of Exonuclease I (New England Biolabs) at 37°C for 60 min, followed by inactivation of 15 min at 80°C. Purified samples were prepared for sequencing using the BigDye 3.1 terminator (Applied Biosystems) as instructed by the manufacturer. The sequencing reaction was as follows: initiating step of 96°C for 1 min, 25 cycles of 96°C for 10 s, 53°C for 5 s, 60°C for 4 min. Sequence samples were purified with the Millipore Multiscreen plates (Millipore, USA) with Sephadex G-50 Superfine Sepharose (Amersham Biosciences, Sweden). Electrophoresis was performed with an ABI 3730 DNA Analyzer (Applied Biosystems) and base calling using the Sequence Analysis 5.2 software (Applied Biosystems). Initial analysis was carried out in Variant Reporter 1.0 software (Applied Biosystems) and the reported results were checked by Sequencher 4.1.4 software (Gene Codes, USA).

### Replication by Sequenom Genotyping

Fourteen SNPs covering the length of the gene (indicated by red rectangles in Figure 2) were included in a single Sequenom iPLEX. The purpose of the Sequenom replication was to increase the sample size to gain reliability of analyses. The assay design and the genotyping were performed with Sequenom MassArray system at the FIMM Technology Centre, University of Helsinki. The Technology Centre performed routine quality control steps to ensure high quality of the genotyping.

### Quality Control

Genotyping results from all methods were tested for deviations from the Hardy–Weinberg equilibrium (*p* < 0.05), and none were discovered in the controls. Data on three individuals were removed due to unresolved discrepancy between the sequenced and the genotyped results and data from 11 individuals were removed due to >10% failed genotyping by the Sequenom (Table S1 in Supplementary Material).

### *In Silico* Analysis of Functionality

Ensemble Variant Effect Predictor and Human Splicing Finder 3.0 online softwares were used to assess the consequences of the five intronic SNPs of interest (29). RNAsnp by Center for non-coding RNA in Technology and Health (RTH) was the final online software that was applied to detect possible local RNA secondary structure changes, which might be introduced by exonic SNPs and could lead to changes in posttranscriptional processes of an otherwise functional gene (30, 31). Mode 2 of the program was used, as it is applicable for large mRNAs (>1,000 nucleotides). Following the suggested limits, a *p*-value <0.2 was considered indicative of an SNP induced change in secondary structure.

### The Relative Extended Haplotype Homozygosity

To predict selection pressure toward the discovered haplotype and pinpoint its structure, we completed a REHH analysis using the associating rs2287845 as a focal SNP (32). REHH was conducted in R following the developer’s instructions to reveal the evolutionary selection pressures underlying the haplotype structure. All Sanger sequenced individuals (initial cohort and replication cohort *N* = 213) were used for REHH and missing genotypes were imputed using fastPHASE software (33, 34).

### Statistical Analyses

The results of the complement SNP genotyping were analyzed in PLINK (35). The *C3* sequencing results were analyzed for association in gPLINK and PLINK. Single SNP association to disease was evaluated by Fisher’s Exact test and results were confirmed in Haploview (36). Haplotype analysis was conducted with the Haploview software (36). Association analysis of individual SNPs and the discovered haplotype blocks was done using χ^2^ test in the Haploview program.

## Results

### SNP Genotypes of Complement Genes

To analyze potential associations between PE and complement genes, we genotyped selected SNPs in 17 genes coding components of the complement system. No differences were observed in 64/72 SNPs between 259 PE women and 426 non-PE controls (data not shown). Out of the remaining eight SNPs, three associated to *C3* in genotypic model analysis and in *C3*, rs2230204, and rs2230205 were associated after permutation. rs2230204 in the proximity of *C3* exon 14 was most likely to have an independent effect (likelihood ratio test: χ^2^ = 5.1, df = 1, *p* = 0.024).

### *C3* Sequences

In the *C3* promoter, exons, and flanking introns, a total of 43 SNPs were identified in severe PE women, non-PE controls, or both (Figure 2). rs200046246 is located in a predicted transcription factor-binding site. It is a missense SNP that causes an amino acid change K779R. It was predicted by SIFT and POLYPHEN2 to be well tolerated and benign, apparently, because there is no change in the charge of the amino acid (both lysine/K and arginine/R are basic residues). Other variants were either synonymous or intronic. Six SNPs were independently associated to severe PE. rs190390034 (−39 from exon 2) had the strongest association with a predisposing effect with minor allele T [χ^2^ = 7.72, *p*-value = 0.005; OR = 7.627 (95% CI 1.442, 40.350)] (Figure 2). While we had appropriate power to assess three of six SNPs with single SNP association: rs2287845 [minor allele frequency (MAF) = 0.426, *p* = 0.038, OR = 1.158, 95% CI = 1.009, 1.331], rs366510 (MAF = 0.426, *p* = 0.039, OR = 1.158, 95% CI = 1.008, 1.330), and rs2287848 (MAF = 0.426, *p* = 0.041, OR = 1.155, 95% CI = 1.006, 1.327), the associations of the remaining three SNPs are only suggestive (Table 2).

**Table 2:** **Six non-coding single nucleotide polymorphism (SNP) have independent allelic association to severe preeclampsia**.

Genomic position (Build 38)	SNP	Minor allele frequency in cases/controls	*N*	χ^2^	*p*-Value	Odds ratio (CI95)
19:6686493	rs2241391	0.023/0.014	1,694	4.071	0.044	1.73 (1.009, 2.966)
19:6696331	rs2287848	0.441/0.405	1,693	4.163	0.041	1.155 (1.006, 1.327)
19:6696586	rs2287845	0.441/0.405	1,693	4.328	0.038	1.158 (1.009, 1.331)
19:6697818	rs366510	0.441/0.405	1,693	4.283	0.039	1.158 (1.008, 1.330)
19:6719442	rs190390034	0.078/0.011	123	7.72	0.005	7.627 (1.442, 40.35)
19:6720961	rs183805948	0.031/0	123	5.734	0.017	NA

*CI95, 95% confidence intervals; NA, not available (the variant was not observed in controls)*.

**Figure 3:**
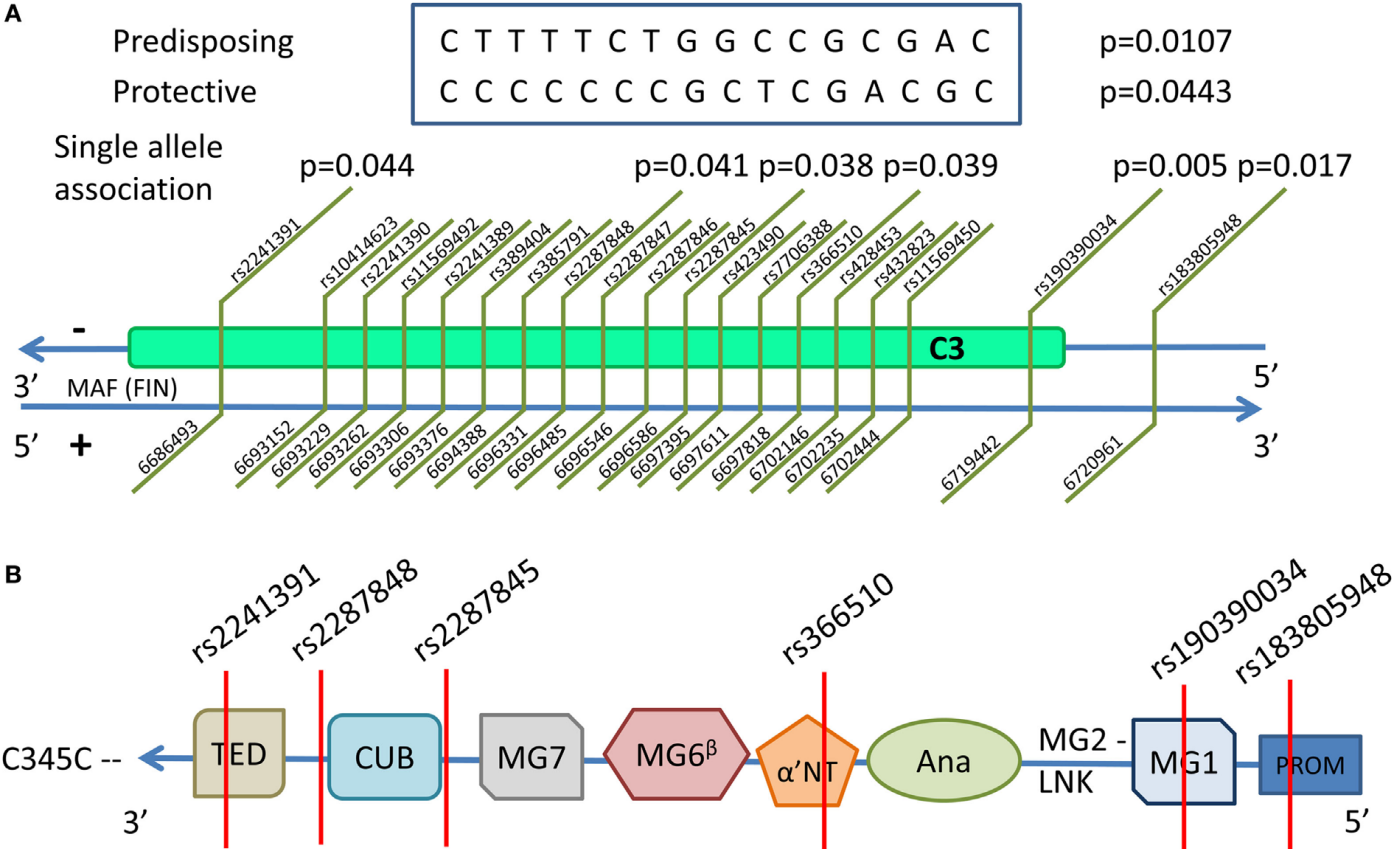
***C3* is located in chromosome 19 in the reverse strand**. The discovered single nucleotide polymorphism (SNP) associations, their positions, and associating haploblocks are depicted in **(A)**. The associating SNPs and their locations in the domains of the functional C3 are shown in the schematic structure in **(B)**. All associating SNPs are located in introns with the exception of rs183805948, which is located in the PROM. The domains are TED—thioester-containing domain (C3d); CUB—complement C1r/C1s, Uegf, Bmp1; MG1–7—macroglobulin domains 1–7; α'NT—N-terminal region of the cleaved α-chain in the linker domain; Ana—anaphylatoxic region (C3a); LNK—β-strand of the linker domain; PROM—promoter region.

A set of alleles spanning from 5' intron proximal to exon 18 to the 5' intron proximal to exon 25 and consisting of 16 SNPs was found to be associated with severe PE. One haplotype showed an association in a protective (frequency in severe PE women and non-PE controls 0.275 and 0.366, respectively, *p* = 0.044), and another one in a predisposing manner (frequency in severe PE women and non-PE controls = 0.182 and 0.094, respectively, *p* = 0.011) (Table 3). Mapping the 16 SNPs contributing to the haplotype along the *C3* gene showed that the tagging SNP rs2287845 is located 7bp in 3' direction of exon 22, which corresponds to the alpha-chain of the gene product at the edge of MG7 and directly before (5') the CUB domain (Figure 3). The proposed haplotype spans across the middle of the gene from the 3' intron of exon 18 situated at the 5' end of the ANA domain right before the α'NT domain and at 5' direction reaching past the CUB and into the TED domain. Three of the SNPs in the haplotype (rs406514, rs11569450, and rs432823) are located in the introns flanking exon 18, which codes for the α'NT domain. The regulatory microsatellite region was not associated with severe PE.

**Table 3:** **The region covered by haploblock 2 had two haplotypes of 16 SNPs with suggestive association to preeclampsia**.

Haploblock 2	Frequency cases/controls	Proposed effect	χ^2^	*p*-Value	Odds ratio (*t*, Wald test)
CCCCCCCGC**T**CGACGC	0.274/0.366	Protective	4.047	0.044	0.628 (6.08)
CTTTTCTGG**C**CGCGAC	0.182/0.094	Predisposing	6.511	0.011	2.110 (4.72)

**Figure 4:**
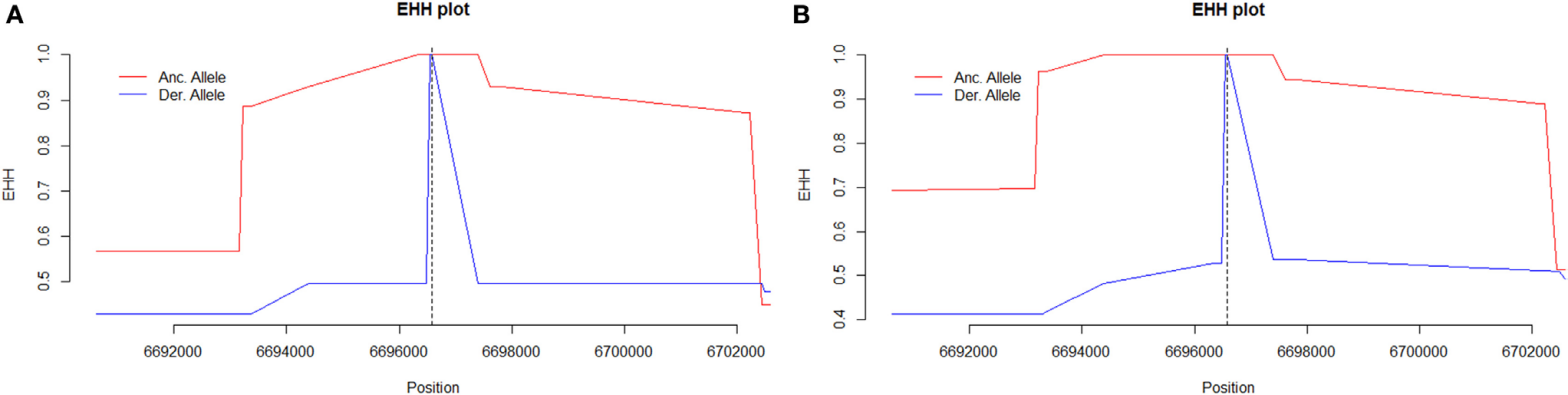
**The extended haplotype homozygosity (EHH) diagram of (A) women with severe preeclampsia (PE) and (B) non-PE controls using rs2287845 as a focal single nucleotide polymorphism (SNP) representative of the haploblock structure**. The ancestral allele curve (in red) is close to 1 for a long stretch in non-PE controls particularly in the 5' upstream direction indicating that the haplotype surrounding the focal SNP (indicated by severed vertical line) is under selection in this cohort. Major allele (ancestral, in red) C, minor allele (derived, in blue) T.

*The focal SNP rs2287845 in relative extended haplotype homozygosity analysis (Figure 4) is indicated in bold*.

### *In Silico* Functional Analysis

Human Splicing Finder 3.0 found splicing motives to be influenced by four of the associating *C3* SNPs: rs190390034, rs2287848, rs366510, and rs2241391. rs366510, rs2241391, and rs190390034 were predicted to create an exonic splicing enhancer (ESE) site within an intron, whereas rs2287848 was found to create a novel ESE site as well as to cause an alteration of an existing ESE site. In RNAsnp, rs190390034 had a *p*-value of 0.029 indicating a disruption in local RNA folding and rs2241391 had a *p*-value of 0.169 indicating a likely disruption in local RNA folding. The associating SNPs within the haploblock region (rs2287845, rs366510, and rs2287848) had RNAsnp *p*-values >0.2 indicating no significant structural change caused by these variants.

### The Relative Extended Haplotype Homozygosity

The tagging SNP rs2287845 was associated with an extended haplotype to the 5' direction on the *C3* gene suggesting that this structure with tight linkage disequilibrium in the middle of the gene results from a positive selection pressure (Figure 4).

## Discussion

In this study, we have described genetic variants of the key complement component C3 in women with severe PE and control women with non-PE pregnancies. We identified a 16-nucleotide haplotype signature in the highly conserved middle region of the maternal *C3* gene with three associating SNPs (rs2287845, rs366510, and rs2287848) that could influence susceptibility to severe PE.

The three *C3* SNPs that associate with severe PE are located in the area of most intense linkage disequilibrium in the middle of the associating haploblock. The discovery of predisposing SNPs within the haploblock structure supports a possible functional role for the haploblock-encoded gene product. Furthermore, rs190390034 is an intronic variant 38 bp 5' downstream of exon 2 that is in tight linkage disequilibrium with the haploblock structure in the middle of the gene. rs190390034 has a perfect D' score of 100 for the tagging SNP rs2287845 (Figure 2). Among 43 observed SNPs, rs190390034 had the best allelic association with severe PE. While suggestive due to insufficient sample size, the independent association of rs190390034 with severe PE supports the role of the haploblock in severe PE. With the available genotypes for the remaining two of the six associating SNPs within *C3* we were underpowered due to insufficient sample size (rs190390034) and rarity of the variants (rs183805948 and rs2241391). Therefore, the latter results should be interpreted with caution.

In support of the causality of changes in the haploblock 2 area of the *C3* gene, two of the three associating SNPs within the haploblock described in our study have been implicated in prior studies in other phenotypes. rs2287845*C has been shown to be significantly associated with overall survival and prognosis in patients with early stage non-small cell lung cancer after surgical resection (37). rs366510 is a probable splicing variant that has been linked to asthma and related phenotypes in two independent studies (38, 39). None of these studies looked for a haplotype association within *C3*. Intronic variants causing *C3* splicing mutations have been described in patients with C3 deficiency due to exon deletion from the C3 mRNA (40, 41). The current study is according to our knowledge the first one to show a disease association in C3 with a haplotype-based mechanism instead of the conventional single SNP association. Indeed, it has become apparent that while mutations leading to changes in amino acid sequences are readily detected, more subtle changes in gene regulatory elements are most likely accountable for much of the phenotypic variation we observe in complex diseases (42, 43). Such regulatory features remain cryptic in analyses at the translational and posttranslational level.

In other genes, intronic disease associations with causative splicing defects have been described (44, 45). In the field of reproductive immunology in a set-up similar to our study, an intronic haplotype was found to result in IL-10 secretion changes in women with idiopathic recurrent miscarriage (46). Furthermore, it was recently shown that mechanical nucleosome binding occurs even on top of genes (47). Therefore, it is possible that non-coding SNPs may have an important regulatory role, e.g., by influencing DNA folding.

Complement C3 plays a central role in a successful pregnancy. Inappropriate complement activation may play a role in the initial stages of PE pregnancies contributing to inadequate placentation or placental dysfunction. The anecdotal reports of success of eculizumab in the treatment of a full-blown disease indicate that the complement system is also involved in the later stages of the disease possibly by generating inflammation or tissue damage (22, 48). Problems may emerge if disturbances in the removal of ischemic or injured placental components by complement and phagocytes occur (49). Lack of functioning C3 in mice led to fewer pregnancies and to a higher fetal reabsorption rate, while fetal and placental weights were lower (50). On the other hand, C3-mediated over-activation of the complement system was shown to induce hypertension following placental ischemia in rats (51). Furthermore, complement activation at the feto-maternal interface of Crry^−/−^ mice that lack a key complement regulator was shown to cause fetal loss. The embryos were rescued when Crry^−/−^ mice were bred to C3^−/−^ mice (52). These observations underline the importance of balanced activation and regulation of the complement system for a healthy pregnancy.

Because C3 activation by the C3 convertases requires extensive conformational changes and translocation of the CUB/TED and α'NT domains (17), protein changes caused by variants in the middle of the *C3* gene may hinder binding of factor B to C3b causing the C3 convertase to function inefficiently (Figure 3). If the haplotype described here has an effect on C3 function as suggested by its critical location, it is possible that C3 activation in the individuals with the protective haplotype is properly regulated. Thereby, the extravillous trophoblasts (EVTs) invading the maternal tissue during placentation would not encounter a vigorous complement attack. Thus, they could successfully remodel the uterine spiral arteries resulting in a non-PE pregnancy with a healthy blood flow and placental development (9). Concurrently, the predisposing haplotype may result in an increased level of complement activation as indicated by increased factor B (Bb) levels early in the pregnancy. Complement attack could compromise the EVT invasion and consequently the placental function resulting in an increased occurrence of severe PE (11, 53).

*C3* promoter activity is dependent on the dinucleotide repeat polymorphism GF100472 such that the longer the CA repeat region, the lower the transcriptional activity of *C3* (54). A shorter repeat has shown protective effect against mesial temporal lobe epilepsies and febrile seizures. However, in the present study, we did not find any indication of association of the CA-repeat to severe PE in a small patient cohort.

The functional polymorphism rs2230199 in *C3* is known as the slow/fast mutation that influences C3 protein mobility in electrophoresis gels. C3F has been described as predisposing to PE (55), but we did not find any association of C3F with severe PE. Our result concurs with an early study of C3 allotypes that did not find association of C3F with PE (56).

The REHH analysis shows that the haploblock structure in the middle of *C3* is tightest for the ancestral rs2287845 allele in non-PE controls suggesting that the structure results from a positive evolutionary selection pressure (Figure 4B). A similar pattern is observed for the ancestral allele of rs2287845 in PE women. However, in severe PE, the haploblock structure disintegrates noticeably sooner than in controls, indicating a loosened force of active selection (Figure 4A). It would follow that due to a possible regulatory feature caused by seemingly benign variants that have been introduced into the middle of *C3*, the risk for severe PE increases, while haplotypes with the ancestral genotype are protective from severe PE and, accordingly, under stronger positive natural selection.

The heterogeneity of PE is reflected in the comparison of our results to another recent study. Wu et al. found that rs698090 in *MASP1* is associated to late-onset but not to early-onset PE, and nominally to severe PE in a Chinese population (57). In our initial genotyping, we did not find allelic or genotypic association of rs608090 to severe PE. It is possible that different complement pathways contribute to early-onset PE and late-onset PE and the mechanism of these varying associations merit more studies.

Targeting gene regulatory effects may provide new opportunities for PE risk assessment and diagnosis, maybe even future drug development (58). The reported results reveal significant differences between PE and healthy pregnant women but the roles of individual SNPs should be considered suggestive and treated with caution. With further studies to confirm our findings, assessing *C3* genetic polymorphisms may be developed as a tool to find patients with the highest risk of severe PE.

## Ethics Statement

All subjects provided a written informed consent in accordance with the Declaration of Helsinki. Study protocols were approved by the local Ethical Committees, specifically, for the FINNPEC study, ethical approval has been obtained from the Coordinating Ethics Committee, Hospital District of Helsinki and Uusimaa, for the Finnish population based pre-eclampsia cohort was approved by the ethics committee of the Finnish Red Cross Blood Service and by the Ministry of Social Affairs and Health and for Southern Finland pre-eclampsia study was approved by the Ethics Committee of the Department of Obstetrics and Gynaecology at Helsinki University Central Hospital.

## Author Contributions

AIL designed the study with SM and HL. AIL and TK designed part of the primers for sequencing, AIL supervised laboratory work, sequenced all samples from patients and some controls, read the results, conducted association analyses of the *C3* sequencing project, performed *in silico* analyses, and drafted the manuscript. TK conducted laboratory work for the regulatory regions branch of the study, analyzed these data, and participated in main data analysis. VH designed the complement genotyping Sequenom chip. PO analyzed the genotyping data. LK performed and interpreted the REHH analysis with AIL and PS. PV described patient cohorts. HL, SH, EK, JK, KK, and AP form the board of the FINNPEC cohort and are responsible for the clinical data and biological samples used in this study. LH provided samples and is with HL responsible for the clinical data of the Finnish population-based preeclampsia cohort. HL provided the samples and clinical data of The Southern Finland preeclampsia cohort. All the authors collaborated in drafting the manuscript and accepted the final version of the manuscript.

## Conflict of Interest Statement

This research was conducted in the absence of any commercial or financial relationships that could be construed as a potential conflict of interest.
