# Supplementary material for: Analysis of Complement C3 Gene Reveals Susceptibility to Severe Preeclampsia
Source: Front Immunol. 2017 May 29;8:589. doi: 10.3389/fimmu.2017.00589 (PMC5446983; doi:10.3389/fimmu.2017.00589)
Supplement: Supplementary file 1 [file table_1.docx]

S1. The number of women from each study cohort studied per diagnosis in each stage of the analysis.

PE=pre-eclampsia, C=controls, SPE=severe pre-eclampsia.

| Method | Groups | Southern Finland Cohort | Finnish population based pre-eclampsia cohort | FINNPEC |
| --- | --- | --- | --- | --- |
| Complement chip screening | PE | 67 | 204 | na |
|  | C | 45 | 404 | na |
|  | Excluded | 9 | 26 | na |
| *C3* Sequencing | SPE | 32 | na | na |
|  | C | na | na | 95 |
|  | Excluded | na | na | 4 |
| Microsatellite regulatory region | SPE | 32 | na | na |
|  | C | na | na | 95 |
|  | Excluded | na | na | 3 |
| Replication by sequencing | SPE | na | na | 95 |
|  | C | na | na | na |
|  | Excluded | na | na | na |
| Replication by Sequenom (incl. all previously listed FINNPEC individuals) | SPE | na | na | 705 |
|  | C | na | na | 960 |
|  | Excluded | na | na | 14 |
| REHH | SPE | 32 | na | 91 |
|  | C | na | na | 91 |
|  | Excluded | na | na | na |
