# Supplementary material for: Analysis of Complement C3 Gene Reveals Susceptibility to Severe Preeclampsia
Source: Front Immunol. 2017 May 29;8:589. doi: 10.3389/fimmu.2017.00589 (PMC5446983; doi:10.3389/fimmu.2017.00589)
Supplement: Supplementary file 2 [file table_2.docx]

S2. List of the genes and variants that were included in the primary complement genotyping SNP. 72/93 SNPs had minor allele frequency of >0.05 and were included in the analyses. 685/720 individuals had genotyping success of >90% and were included in the analyses.

| Chromosome | Gene | RSID |
| --- | --- | --- |
| 1 | MASP2 | RS2273347 |
| 1 | MASP2 | RS1033638 |
| 1 | MASP2 | RS1782455 |
| 1 | MASP2 | RS12711521 |
| 1 | MASP2 | RS3765900 |
| 1 | FCN3 | RS4970521 |
| 1 | FCN3 | RS10794501 |
| 1 | THBS3 | RS35154152 |
| 1 | THBS3 | RS914615 |
| 1 | CFH | RS800292 |
| 1 | CFH | RS1061147 |
| 1 | CFH | RS2274700 |
| 1 | CFH | RS1329428 |
| 3 | MASP1 | RS698090 |
| 3 | MASP1 | RS698092 |
| 3 | MASP1 | RS3105782 |
| 3 | MASP1 | RS1357134 |
| 3 | MASP1 | RS850307 |
| 3 | MASP1 | RS6783637 |
| 3 | MASP1 | RS7624953 |
| 5 | THBS4 | RS3813667 |
| 5 | THBS4 | RS423906 |
| 6 | CFB | RS641153 |
| 6 | CFB | RS537160 |
| 6 | CFB | RS541862 |
| 6 | CFB | RS4151658 |
| 6 | CFB | RS2072633 |
| 6 | THBS2 | RS7382711 |
| 6 | THBS2 | RS12178180 |
| 9 | FCN2 | RS3124952 |
| 9 | FCN2 | RS3811140 |
| 9 | FCN2 | RS7865453 |
| 9 | FCN2 | RS17514136 |
| 9 | FCN2 | RS3128624 |
| 9 | FCN2 | RS7037264 |
| 9 | FCN2 | RS12684723 |
| 9 | FCN2 | RS17549193 |
| 9 | FCN2 | RS7851696 |
| 9 | FCN1 | RS1071583 |
| 9 | FCN1 | RS2274845 |
| 9 | FCN1 | RS2989722 |
| 9 | FCN1 | RS10858293 |
| 9 | FCN1 | RS10117466 |
| 9 | FCN1 | RS10120023 |
| 9 | FCN1 | RS2989727 |
| 10 | MBL2 | RS5030737 |
| 10 | MBL2 | rs1800450 |
| 10 | MBL2 | Rs7096206 |
| 10 | MBL2 | Rs11003125 |
| 11 | SERPING1 | RS3758918 |
| 11 | SERPING1 | RS1005510 |
| 11 | SERPING1 | RS1557522 |
| 11 | SERPING1 | RS4926 |
| 13 | FLT1 | RS9554314 |
| 13 | FLT1 | RS7326277 |
| 13 | FLT1 | RS7993418 |
| 13 | FLT1 | RS2296189 |
| 15 | THBS1 | RS2228261 |
| 15 | THBS1 | RS2292305 |
| 15 | THBS1 | RS2228263 |
| 19 | C3 | RS17030 |
| 19 | C3 | RS10402876 |
| 19 | C3 | RS423490 |
| 19 | C3 | RS366510 |
| 19 | C3 | RS2230205 |
| 19 | C3 | RS2230204 |
| 19 | C3 | RS1047286 |
| 20 | THBD | RS1042580 |
| 20 | THBD | RS1042579 |
| 23 | CFP | RS1048118 |
| 23 | CFP | RS8177079 |
| 23 | CFP | RS909523 |
