# Supplementary material for: Analysis of Complement C3 Gene Reveals Susceptibility to Severe Preeclampsia
Source: Front Immunol. 2017 May 29;8:589. doi: 10.3389/fimmu.2017.00589 (PMC5446983; doi:10.3389/fimmu.2017.00589)
Supplement: Supplementary file 3 [file table_3.docx]

| S3. Sequencing and fragment analysis primers and details. Primers on the coding strand (-) are in upper case while primers in the non-coding (+) strand are in lower case. All primers were created using the software Primer3 (<http://bioinfo.ut.ee/primer3-0.4.0/>). | | | | |
| --- | --- | --- | --- | --- |
|  | Left Primer | Right Primer | Product size (bp) | Annealing temperature (C°) |
| (CA)_n_ repeat | FAM-ATGGGAGGAAGACCACCTTT | CCCCTCACTTACCCTTGTCA | 245-259 | 67 |
| Promoter | GGGAGCCTCCTTGGAAAATA | AGCCCTGTTTGTGGGTAGAG | 852 | 63 |
| Exon1 | CTGCTCACTCCTCCCCATC | AAATGTCTGCTTCCACCCC | 200 | 65 |
| Exon2 | GAGGACTGGCGTCTCACATC | ggaggggctcaggaggag | 328 | 65 |
| Exon3-4 | CAAGATCCGGAAGCTGGAC | TTGCCTCTCCTAAGCCTGTG | 446 | 65 |
| Exon5-7 | AGCTGAGAGGCTAAGCCCAG | GTCTTCACCTGGTCCCTCAC | 579 | 65 |
| Exon8-9 | GGAGATCCCATTCTCCAGG | CTTCTGACCTGGTCTCCCC | 450 | 64 |
| Exon10-11 | GGAGGTCTAATCCTGAGGGG | GAACCCCTGTACCGTCTTCC | 479 | 65 |
| Exon12-13 | caccaattcccaggtctcag | agacagttgagagacagagaggg | 807 | 63 |
| Exon14 | CATCCCAGGCACTCCTCTC | CTCCAGTCCCACCCACCTC | 299 | 65 |
| Exon15-16 | GTGGGGTCATTTGGGAAGAG | TCCCCTCCTCCCTCTCTG | 652 | 65 |
| Exon17 | GAAGTCCTCCCTGGGGTC | TCCCTCCTCAGACAGGAGTC | 357 | 59 |
| Exon18-19 | ccaactcctggcctcaag | ATGACACTCAGACACCCTGG | 630 | 65 |
| Exon20-21 | AAGAGCTGAGACCCAGGAGC | GAAGACCAGGAGCCCTCTC | 583 | 65 |
| Exon22-23 | TGCTGACCATCTGTGTGTCTG | ATGAGATGGAATTTGGCTCC | 420 | 65 |
| Exon24 | AACCCTTTTCACGCCACC | GGGATCTTAGGGGAGGGATG | 344 | 65 |
| Exon25-26 | CTGTCCCCTCTCTGCACC | CTCTCGTGTTCATCCTGCG | 726 | 65 |
| Exon27 | GATGACTGCCATGTGTGGAC | CAAGGTGGCTGTGCTCTG | 222 | 65 |
| Exon28-29 | TCCTTACTAACGTGACAGCAATG | CTAGGAGGCCAGTGGGAAG | 906 | 65 |
| Exon30-33 | GATGTCCCAGCTCTGATTTG | ACTTGGAAAGTACTGAATATCATGG | 954 | 63 |
| Exon34-35 | TCCTTGTCCAGGAACAGACC | CAGCCAGATAGAGGTCAGGG | 423 | 65 |
| Exon36 | CCCAAGACAATGCTGGACTC | CCCCACAATTCATATATACCTGG | 246 | 65 |
| Exon37-38 | TGGTCTTTGGAGGGAGGC | CAACCACACCTACCACCCAC | 504 | 65 |
| Exon39-41 | GTGCCCCTCATGGTCAAC | GGCAAAGAACTCCAGACACG | 697 | 65 |
|  |  |  |  |  |
|  |  |  |  |  |
