# Supplementary material for: Analysis of Complement C3 Gene Reveals Susceptibility to Severe Preeclampsia
Source: Front Immunol. 2017 May 29;8:589. doi: 10.3389/fimmu.2017.00589 (PMC5446983; doi:10.3389/fimmu.2017.00589)
Supplement: Supplementary file 4 [file table_4.docx]

S4. The 43 SNPs that were observed in *C3* (See Figure 2).

| Order 5’ -> 3’ | RSID | Genomic position (GRCh38) |
| --- | --- | --- |
| 1 | rs17030 | 6677978 |
| 2 | rs344555 | 6679349 |
| 3 | rs7951 | 6681980 |
| 4 | rs45532534 | 6682103 |
| 5 | rs11666133 | 6686410 |
| 6 | rs2241391 | 6686493 |
| 7 | rs11665922 | 6686567 |
| 8 | rs237554 | 6686648 |
| 9 | rs3745568 | 6690602 |
| 10 | rs375600369 | 6690629 |
| 11 | rs10414623 | 6693152 |
| 12 | rs2241390 | 6693229 |
| 13 | rs11569492 | 6693262 |
| 14 | rs2241389 | 6693306 |
| 15 | rs389404 | 6693376 |
| 16 | rs385791 | 6694388 |
| 17 | rs2287848 | 6696331 |
| 18 | rs2287847 | 6696485 |
| 19 | rs2287846 | 6696546 |
| 20 | rs2287845 | 6696586 |
| 21 | rs423490 | 6697395 |
| 22 | rs77063881 | 6697611 |
| 23 | rs366510 | 6697818 |
| 24 | rs428453 | 6702146 |
| 25 | rs432823 | 6702235 |
| 26 | rs11569450 | 6702444 |
| 27 | rs200046246 | 6702489 |
| 28 | rs406514 | 6702587 |
| 29 | rs558232596 | 6707655 |
| 30 | rs144432231 | 6707902 |
| 31 | rs2230205 | 6709693 |
| 32 | rs2230204 | 6709837 |
| 33 | rs2230203 | 6710771 |
| 34 | rs10411506 | 6710937 |
| 35 | rs11085197 | 6713164 |
| 36 | rs1047286 | 6713251 |
| 37 | rs2230201 | 6713280 |
| 38 | rs11569571 | 6714024 |
| 39 | rs2230199 | 6718376 |
| 40 | rs190390034 | 6719442 |
| 41 | rs11569569 | 6720709 |
| 42 | rs183805948 | 6720961 |
| 43 | rs146998274 | 6721227 |
